# Paradigm shift in the application of patient contact shielding: a balancing act between tradition and progress

**DOI:** 10.1007/s00330-025-12006-0

**Published:** 2025-09-18

**Authors:** Till Schürmann, Friederike Lang, Annika Jakobi, Alexander Rau, Jakob Weiss, Katharina Mueller-Peltzer, Kristin Goller-Bruchmann, Wibke Uller, Christopher L. Schlett, Fabian Bamberg, Martin Fiebich, Thomas Stein

**Affiliations:** 1 Department of Diagnostic and Interventional Radiology, Medical Center—University of Freiburg, Faculty of Medicine, University of Freiburg, Freiburg, Germany; 2 Department of Neuroradiology, Medical Center—University of Freiburg, Faculty of Medicine, University of Freiburg, 79106 Freiburg, Germany; 3 University of Applied Sciences Mittelhessen, Faculty of Life Science Engineering, Institute of Medical Physics and Radiation Protection, Gießen, Germany

**Keywords:** Computed Tomography, Interventional radiology, Patient shielding, Projection radiography, Radiation protection

## Abstract

**Objectives:**

Despite recommendations and guidelines on patient contact shielding in X-ray imaging, substantial uncertainties remain in clinical practice, particularly concerning computed tomography (CT) examinations and vulnerable groups such as pediatric and pregnant patients. This study identifies gaps in existing recommendations and offers a comprehensive statement of the actual risks and benefits associated with patient shielding.

**Materials and methods:**

A systematic literature search was conducted using Google Scholar and PubMed, alongside current national and international guidelines. Our special report focused on patient shielding in projection radiography, interventional radiology, and CT, with special emphasis on vulnerable patient groups sensitive to radiation exposure.

**Results:**

Current research lacks robust, evidence-based data comparing the benefits and risks of patient shielding, especially in CT. In projection radiography and interventional radiology, patient shielding offers minimal benefits and may inadvertently increase radiation exposure due to interference with automatic exposure control or necessitate repeated examinations. This issue is particularly addressed in pediatric and pregnant patients. In CT, the benefits and risks are more complex, with substantial research gaps hindering informed decision-making.

**Conclusion:**

Traditional and generalized recommendations for patient contact shielding do not adequately account for technological advancements and individual patient needs. The use of patient shielding should be reconsidered on a case-by-case basis, guided by evidence-based research. There is an urgent need for clinical studies to assess the benefits, and in particular the risks in real-world settings, facilitating the development of precise patient-specific guidelines.

**Key Points:**

***Question*** *While patient shielding can increase radiation dose due to interference with automatic exposure controls, uncertainties persist regarding patient shielding in X-ray imaging practices*.

***Findings*** *There is marginal evidence of the clinical risks of patient shielding, and urgent needs exist for patient-specific evidence-based shielding guidelines*.

***Clinical relevance*** *By critically evaluating the ambiguous guidelines on patient shielding and highlighting the lack of evidence-based risks of patient shielding, this study argues for individualized, evidence-based practices to improve patient safety in clinical radiology*.

## Introduction

Patient contact shielding was introduced about seventy years ago to protect sensitive structures such as the gonads from genetic effects associated with radiation exposure [1, 2]. While little has changed in the use of patient shielding since its introduction [3], technological advances in X-ray diagnostics (e.g., precise collimation, shorter exposure times, automatic exposure control (AEC), or digital processing) have significantly improved image quality while reducing patient dose [1, 4–6].

**Fig. 1:**
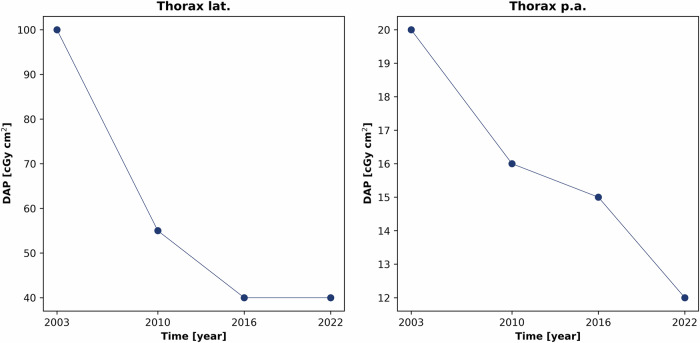
Reduction of the diagnostic reference levels (DRLs) for thoracic projection images (lateral and posterior-anterior) of an adult from 2003 to 2022, illustrated by the decreasing dose area product (DAP) [7–10]

**Fig. 2:**
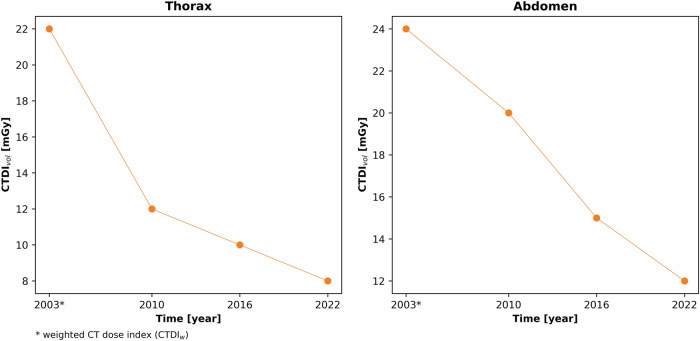
Reduction of the diagnostic reference levels (DRLs) for CT imaging of the thorax and abdomen in adults from 2003 to 2022, illustrated by the respective decreasing volume CT dose indices (CTDI_vol_) [7–10]

The advancements in X-ray technology are, e.g., reflected in the decreased German diagnostic reference levels (DRLs). Since 2003, DRLs for chest radiographs have decreased by 60% for lateral projections and 40% for posterior-anterior projections [7–10], with similar trends in pediatric DRLs (Fig. 1). CT examinations show continuous DRL reductions from 2003 to 2022, illustrating the success of dose reduction technologies by the decrease of the volume CT dose index (CTDI_vol_) [7–10] (Fig. 2).

International societies are reassessing the use of patient shielding due to technological advances and reduced radiation exposure [1, 11]. Since the establishment of AEC, improperly positioned patient shielding can negatively affect X-ray systems, potentially increasing radiation or influencing diagnostic accuracy [12, 13]. Thus, the benefits of patient contact shielding are accompanied by corresponding risks [14, 15], leading to debates among associations [1, 15–17].

Concurrently, advances in the understanding of human tissue radiosensitivity have led the International Commission on Radiological Protection (ICRP) to reduce the tissue weighting factor for gonads from 0.25 to 0.08 over time since 1977 [18–20], as no significant increases in heritable radiation-induced diseases have been documented [20]. Another essential radiosensitive organ is the breast, which has shown significant fluctuations in the tissue weighting factor over time, from 0.15 to 0.05, with an increase to 0.12 [18–20] due to recent epidemiological findings of the cancer incidence [21].

Nevertheless, clinical practice reveals ongoing uncertainties, particularly regarding the optimization of individual CT examinations, which may vary significantly depending on the clinical indication, patient-specific anatomical and physiological factors, the parameters of the protocol and the manufacturer. Furthermore, vulnerable patient groups sensitive to radiation exposure, such as pregnant women, pediatric patients, and individuals with preexisting conditions, require tailored approaches to minimize radiation. Specific recommendations are often undefined, leaving risk-benefit assessments to users [1, 5]. International recommendations such as those from the American Association of Physicists in Medicine (AAPM) [16], the European consensus recommendation on patient contact shielding [1], the British Institute of Radiology (BIR) [15], or the Swiss Society of Radiobiology and Medical Physics (SSRMP) [17] generally do not recommend the use of patient contact shielding in projection radiography, interventional radiology and CT, even for vulnerable patient groups. Conversely, some European societies, such as the Nordic Society for Radiation Protection [22, 23] or the Commission on Radiological Protection in Germany (SSK) [5], consider shield application based on usability, modality, examination method and workload, but provide limited guidance or leave usage unspecified, particularly for pediatric or pregnant patients.

This special report aims to identify gaps in existing recommendations and to discuss both the benefits and potential risks of patient shielding, especially regarding patient groups with increased sensitivity to radiation.

## Materials and methods

The literature review with narrative synthesis and evidence-based statements includes scientific publications from the Google Scholar and PubMed databases, as well as current national and international guidelines from Europe and the USA, covering the period from 1977 to the end of 2023. It focuses on both the effects of patient shielding in general and on vulnerable patient groups such as children and pregnant patients for projection radiography, interventional radiology, and CT modalities. The term “vulnerable” refers to patient groups with increased radiosensitivity. Reports from medical physics societies and reviews on this topic were also included to capture the spectrum of the current understanding. In addition, the German national DRLs were used as radiation dose indices in the following Tables 1–4 for the risk-benefit assessment to illustrate the different amount of absolute dose used in each examination region and to facilitate the necessity of correct shielding.

**Table 1:** Risk-benefit ratio in terms of possible radiation dose reduction [17] using patient shields in pediatric projection radiography outside the primary beam and the risk of increased radiation exposure or obscuring of the examination

Examination	Mean effective doses of DRL (mSv) [66]	Patient contact shielding	Scattered mean effective dose of the organ to be protected (mSv)^a^	Benefit in the possible radiation dose reduction of the organ to be protected (%)	Risk of obscuring region of interest or radiation dose increase due to patient contact shielding (%)
Thorax a.p.^1^/p.a.^2^ & lat.^3^	0.025–0.070	Gonad shielding	0.00007 [68]	4 [46]	-/-^b^
Thoracic spine a.p.^1^/lat.^3^	0.146–0.207	-/-^b^	-/-^b^	-/-^b^	-/-^b^
Lumbar spine a.p.^1^/ p.a.^2^	0.146–0.420	Breast shielding	0.031–0.054 [69]	77–80 [70]	-/-^b^
	0.146–0.420	Testicle shielding	0.003 [71]	42 [71]	-/-^b^
	0.146–0.420	Ovarian shielding	0.043 [71]	0.4 [71]	-/-^b^
Lumbar spine lat.^3^	0.466	Testicle shielding	0.0008 [71]	9 [71]	-/-^b^
	0.466	Ovarian shielding	0.105 [71]	16 [71]	-/-^b^
Abdomen a.p.^1^/p.a.^2^	0.419	Testicle shielding	-/-^b^	26 [13, 46]	-/-^b^
	0.419	Ovarian shielding	-/-^b^	26 [13]	-/-^b^
Pelvis a.p.^1^/p.a.^2^ & lat.^3^	0.463	Testicle shielding	-/-^b^	26–36 [46]	49–66 [27, 32, 40, 41]
	0.463	Ovarian shielding	-/-^b^	26 [46]	74–98.4 [27, 28, 72] with 63–147 dose increase [13]
Hip joint	0.135	Testicle shielding	-/-^b^	26 [46]	52–66 [27, 32, 41]
	0.135	Ovarian shielding	-/-^b^	26 [46]	66–98 [27, 28, 72]
Extremities	-/-^b^	Gonad shielding	-/-^b^	-/-^b^	-/-^b^

^a^ Calculation of the mean effective dose of the organ to be protected using the current weighting factors of ICRP Publication 103 [20]. ^1^a.p. = anterior-posterior, ^2^p.a. = posterior-anterior, ^3^lat. = lateral

^b^ In the case of gaps (-/-) regarding the benefit or risk of patient contact shielding, the respective institution should weigh up the possible organ equivalent dose or dose reduction by means of phantom scans and the possible risk of past examinations in order to be able to make a distinct decision. For adults, a lower risk for the application of patient shielding can be assumed since the cooperation of adult patients is better than that of pediatric patients. The given dose assessments must be adapted for pediatric patients. The table serves as an orientation and can be used for both adults and pediatric patients

**Table 2:** Typical fetal doses for common X-ray examinations in projection radiography and the risk-benefit ratio for patient shields. There are still gaps in the risk-benefit assessment

Examination	Typical fetal doses (mSv) [45]	Patient contact shielding	Scattered mean effective dose of the fetus to be protected (mSv)^a^ [45]	Benefit in the possible radiation dose reduction of the fetus to be protected (%)	Risk of obscuring region of interest or radiation dose increase due to patient contact shielding (%)
Thorax a.p^1^./p.a.^2^ & lat.^3^	0.001–0.002	Lead blanket	0.001–0.002	-/-^b^	-/-^b^
Thoracic spine a.p. ^1^/ lat.^3^	0.011–0.014	Lead blanket	0.011–0.014	-/-^b^	-/-^b^
Lumbar spine a.p^1^./lat. ^3^	1.5–4.0	Lead blanket	1.5–4.0	Not useful/not applicable	Not useful/not applicable
Abdomen a.p^1^./p.a.^2^	1.4 [69]; 2.3–4.5	Lead blanket	1.4 [69]; 2.3–4.5	Not useful/not applicable	Not useful/not applicable
Hip	4.6	Lead blanket	4.6	-/-^b^	-/-^b^

^a^ Calculation of the mean effective dose of the organ to be protected using the current weighting factors of ICRP Publication 103 [20]. ^1^a.p. = anterior-posterior, ^2^p.a. = posterior-anterior, ^3^lat. = lateral

^b^ In the case of gaps (-/-) regarding the benefit or risk of patient contact shielding, the respective institution should weigh up the possible organ equivalent dose or dose reduction by means of phantom scans and the possible risk of past examinations in order to be able to make a distinct decision

**Table 3:** Risk-benefit ratio in terms of possible radiation dose reduction [17] using patient shields in CT outside the primary beam, and the risk of increased radiation exposure or obscuring of the examination

Examination	Mean effective doses of DRL (mSv) [66]	Patient contact shielding	Scattered mean effective dose of the organ to be protected (mSv)^a^	Benefit in the possible radiation dose reduction of the organ to be protected (%)	Risk of obscuring region of interest or radiation dose increase due to patient contact shielding (%)
Cranium	1.57	Eye lens shielding (in the primary beam path)	-/-^b^	2–70 [46, 73, 74]	-/-^b^
	1.57	Thyroid shielding	0.106 [51]	5 [51]–45 [46]	-/-^b^
	1.57	Breast shielding	-/-^b^	25–70 [51]	-/-^b^
Paranasal sinus	0.17	-/-^b^	-/-^b^	-/-^b^	-/-^b^
Facial skull	0.58	-/-^b^	-/-^b^	-/-^b^	-/-^b^
Chest	3.76	Eye lens shielding	0.32 [55] (organ dose)	31 [55]	-/-^b^
	3.76	Thyroid shielding	0.335 [55]	31 [55]–50 [75]	-/-^b^
	3.76	Breast shielding (in the primary beam path)	-/-^b^	12–57 [46]	-/-^b^
	3.76	Lead blanket for abdomen	0.002 [54]–0.26 [76]	10 [54]–78 [46, 76]	-/-^b^
	3.76	Gonad shielding	0.001–1.54 [77]	56–96 [77]	-/-^b^
Abdomen/Pelvis	8.04	Thyroid shielding	0.001–0.006 [78]	64–87 [78]	-/-^b^
	8.04	Breast shielding	-/-^b^	16–26 [46]	-/-^b^
	8.04	Testicle shielding	0.192 [57]	58 [50]–87 [57]	-/-^b^
	8.04	Ovarian shielding	-/-^b^	-/-^b^	-/-^b^
Extremities	-/-^b^	-/-^b^	-/-^b^	-/-^b^	-/-^b^

^a^ Calculation of the mean effective dose of the organ to be protected using the current weighting factors of ICRP Publication 103 [20]

^b^ In the case of gaps (-/-) regarding the benefit or risk of patient contact shielding, the respective institution should weigh up the possible organ equivalent dose or dose reduction by means of phantom scans and the possible risk of past examinations in order to be able to make a distinct decision

**Table 4:** Typical fetal doses for common X-ray examinations in CT and the risk-benefit ratio for patient shields. There are still gaps in the risk-benefit assessment

Examination	Typical fetal doses (mSv) [45]	Patient contact shielding	Scattered mean effective dose of the fetus to be protected (mSv)^a^ [45]	Benefit in the possible radiation dose reduction of the fetus to be protected (%)	Risk of obscuring region of interest or radiation dose increase due to patient contact shielding (%)
Chest	0.13 [79]–0.16 [46]	Lead blanket	0.13 [79]–0.16 [46]	20 [46]–69 [79]	-/-^b^
Pulmonary angiogram	0.01 [45]–0.77 [80]	Lead blanket	0.01 [45]–1.04 [80]	10 [80]–35 [81]	-/-^b^
Abdomen	2.1–2.4	Lead blanket	2.1–2.4 [82]	Not applicable/not useful	Not applicable/not useful
Pelvis/Hip	9.0–25.0	Lead blanket	9.0–25.0	-/-^b^	-/-^b^

^a^ Calculation of the mean effective dose of the organ to be protected using the current weighting factors of ICRP Publication 103 [20]

^b^ In the case of gaps (-/-) regarding the benefit or risk of patient contact shielding, the respective institution should weigh up the possible organ equivalent dose or dose reduction by means of phantom scans and the possible risk of past examinations in order to be able to make a distinct decision

This statement involved authors and co-authors of various departments and fields in X-ray diagnostics, including experts (medical physicists and physicians) of radiation protection, diagnostic, interventional, and pediatric radiology, as well as neuroradiology with expertise in thoracoabdominal, head, cardiovascular, and emergency imaging.

### Methodology of risk-benefit assessment

Results for projection radiography and CT are summarized in Tables 1–4 in the following sections, where the use of patient shielding in diagnostic imaging is analyzed, using a standardized format to ensure comparability across modalities and patient groups. The first column specifies the anatomical region considered for shielding. The column “Scattered mean effective dose of the organ (or fetus) to be protected (mSv)” indicates the organ’s potential exposure to scattered radiation using the effective dose, which was calculated by the current weighting factors of the ICRP publication 103 [20]. The next column presents the possible radiation dose reduction (%), illustrating shielding effectiveness. The last column highlights potential risks of obscuring clinically relevant structures (percentage of affected cases). Furthermore, the potential dose increase (%) due to the interference with the AEC is indicated with “dose increase” if studies exist for the used patient shield and specific examination region.

## Results

Numerous recent position statements [16, 24], guidelines [22, 25] and actual literature [13, 26–32] have revealed significant concerns regarding the use of patient shielding in X-ray examinations. The controversial debate of the use is still frequently discussed in terms of patient shields that can lead to increased radiation exposure and associated risks for patients. Reasons for this are incorrect exposures, including inappropriate adjustments of the X-ray device due to minor experience, or covering clinically relevant structures, which pose a risk that can lead to repeated exposures. Besides incorrect positioning, the patient’s cooperation is also required to avoid patient motion in examinations that increases the risk of repetition in X-ray imaging [33].

### Projection radiography

#### General projection radiography

Patient shielding in projection radiography (typically lead-rubber aprons or gonadal shields) provides minimal benefit when placed outside the primary beam [34, 35] and is ineffective in shielding intracorporeal radiation [1, 3, 6, 11, 15, 16, 24, 25, 34, 35]. Accurate ovarian shielding is challenging [27, 29, 30, 36], and even when correctly placed, it may obscure diagnostically relevant structures [14, 36]. Accordingly, the risk that lead-rubber aprons or gonad shielding obscures imaging of relevant anatomical structures can lead to repeated imaging and additional exposure for the patient [15, 16, 22, 24, 25].

#### Projection radiography of vulnerable patient groups

In pediatric patients, positioning shields is complex and may lead to repeat imaging due to movement or incorrect placement, especially during pelvic exams [28, 37]. In addition, the interference of patient shields with the AEC of digital X-ray devices used in projection radiography can lead to increased radiation [13]. In Europe, only the guideline of the SSK [5] does not recommend the use of AEC for young children, so that the risk of interference with AEC is primarily present in older children. The studies in projection radiography indicate a lack of awareness regarding the risk of patient shielding unintentionally entering the primary beam [1, 6, 11, 13, 15, 16, 22, 24–26, 34, 38]. Furthermore, hygienic concerns are also mentioned by Gilligan et al [11] since these represent a potential source of infection, especially during pandemic situations, particularly in neonatal and pediatric patients. Table 1 summarizes the benefits of patient shields versus their risk within specific pediatric examination regions. Weighing the benefits in terms of dose reduction and the risk of increased radiation exposure due to patient shields, which possibly comes with covering of examination regions, a decision can be made on the application of patient shielding. However, it must be noted that the averaged effective doses across all age groups and sexes are calculated and listed, while the benefits and risks of the last two columns in Table 1 refer to data of the pediatric projection radiography. Therefore, for the listed effective doses, the stochastic risk is higher for pediatric patients and increases with decreasing age according to Wall et al [39].

In neonatal imaging, shielding can increase radiation when AEC is utilized [13] and obscure relevant anatomy, leading to repeat exposures [28, 40–42]. However, neonatal examinations in the neonatal intensive care unit (NICU) are typically performed without AEC, and exposure settings are manually determined. Shields used in incubator settings pose hygiene challenges and are ineffective due to beam geometry [11]. Del Vecchio et al [43] generally reject neonate shielding that is not applied directly to the body due to its lack of usefulness. The focus should be on reducing the number of exposures through optimized collimation and weight-adapted protocols, which may be more effective in lowering radiation than patient shielding. Under consideration of the current evidence, patient contact shielding should not be used in neonatal imaging.

For pregnant patients, shields offer limited fetal protection, as most fetal exposure is from intracorporeal radiation [1]. Contrary to this issue, the SSK recommends the use of patient shields in clinical practice for pregnant patients [5]. Psychological benefits include a sense of safety and improved patient compliance. Careful positioning is essential to avoid obscuring diagnostic regions of interest or interfering with AEC, which could increase exposure. Conversely, shielding may create a false sense of security or radiophobia [44]. To decide on patient shielding during pregnancy, a risk-benefit assessment (e.g., using Table 2) should be applied, listing fetal doses from standard projection radiography. Uterine doses below 50 mGy are not associated with evidenced effects in any phase of pregnancy [45]. This underscores the need for differentiated risk communication and decision-making in radiological exams during pregnancy. However, Table 2 shows the gaps in this topic, which should be filled with further studies, since no publications exist on this topic yet. In this respect, an overview of the benefits and risks would be desirable in international recommendations for an evidence-based approach in clinical practice.

### Interventional radiology

In interventional radiology, shielding is impractical for most angiographic interventions [46, 47]. Protective equipment may enter the radiation path due to certain angulations, increasing patient dose via AEC. Peripheral shielding offers minimal dose reduction of extrafocal radiation, and intracorporeal radiation cannot be reduced [1]. Due to the large exam area, shielding is not recommended as it may obscure critical regions. At this point, in addition to correct positioning and the necessary immobilization, such as mild sedation, anxiolysis, or, in some cases, sedation administered as needed by anesthesiology staff, the most effective procedure for reducing radiation is the ideal collimation, tailored to the object- and indication. Here, the percentage decrease in field size with a smaller field of view plays a particularly important role in pediatric imaging [5]. Additionally, patient shielding is especially not recommended in pediatric interventions due to hygienic concerns [48, 49].

For CT-guided interventional radiology, data on shielding are limited. CT imaging typically uses higher tube voltages and has a different scatter distribution compared to angiography systems. Furthermore, CT-guided interventions are typically performed with a fixed tube current without AEC and thus, without the risk of dose increase by the AEC. Therefore, if shielding can be safely positioned outside the scan area, it may benefit patients, especially including vulnerable groups such as children or pregnant women. Nevertheless, there is a risk of incorrect positioning inside the scan area, which can result in artifacts. This topic is illustrated in the following CT section. Moreover, hygiene considerations remain important. In addition, the use of sterile drapes during CT interventions makes it difficult to monitor the position of the shield or to determine if it has shifted, further complicating its effective use.

### Computed tomography

Since 2019, there has been a consensus among most international professional societies against the use of patient shielding in CT, also specifically for vulnerable groups [3, 15–17]. The AAPM has taken a pioneering role [16], which was followed by other professional societies [3]. The rejection of patient shields is based on the background that they entail the additional risk of increased radiation exposure due to the interaction of patient shielding with AEC [12]. In addition, shielding can impair the visibility of diagnostically relevant regions and, in the worst case, cause artifacts as reported for the gonads [50], potentially leading to repeat CT scans if images are insufficient for diagnosis. Despite association guidelines, few studies assess the actual clinical risk (see Table 3). Most publications rely on phantom studies, which both support [51, 52] and oppose patient shielding in CT [45, 50, 53, 54].

Aside from the BIR [15] and SSRMP [17] guidelines, the position is less restrictive in Europe. According to the European consensus, patient shielding is permitted in special cases, such as in cone-beam CT [1]. Beyond that, the German guidelines recommend the use of different patient shields for specific types of examinations under certain conditions [5, 47]. The protective equipment can be used in particular for the eye lenses [51, 55], the thyroid gland [51, 56], the gonads [57] and the breast [51] as well as for vulnerable patient groups [52].

#### Criteria for the use of patient shielding in CT

For safe use in CT, shields must be placed outside the scan area and be securely fixed to prevent displacement. If these conditions cannot be guaranteed, shielding should not be used. If visible on the topogram, an unshielded topogram scan is recommended to prevent AEC-based dose increases due to attenuation profiling [58, 59]. Shields can cause increased tube current modulation and artifacts, impairing image quality [12, 50, 53, 54, 60]. A new topogram without shielding is recommended if patient shields are outside the scan area to avoid dose increases from the overranging effect [3, 61].

If patient shields are visible on the topogram near the exam area, considering the overranging effect is crucial. A safe minimum distance of the patient shields from the actual examination area can be derived from various publications [62–64]. Accordingly, the safety distance $d$ can be determined from the desired pitch and collimation, as in the calculation example below, and provides a reliable guide for its adequate placement.

$$d={pitch} \cdot {collimation}$$

For instance, an abdominal CT (pitch = 0.8, collimation =  144 × 0.4 mm) yields

$$d=0.8\cdot 144\cdot 0.4 \; {{\rm{mm}}}=46.08 \; {{\rm{mm}}}$$

This rule of thumb provides a minimum distance between the end of the scanned area and the edge of the shield of ~4.7 cm based on the example calculation of an abdominal CT. This formula ensures shields do not interfere with the AEC while providing protection.

While the aforementioned criteria for the use of patient shielding in CT play a key role in minimizing the risk, a risk-benefit assessment can be conducted using the following section.

#### Risk-benefit assessment of patient shielding in CT

Any patient shield applied can reduce the dose to sensitive organs, as illustrated in Table 3. However, their potential risks must be carefully evaluated, and the risk-benefit ratio for the respective institution must be weighed up according to the examined region. If there is doubt about shield placement in the primary beam or overranging area, it is better not to use shielding, as it may obscure regions or increase radiation dose. The risk varies by manufacturers [12], making it crucial to understand AEC control mechanisms, as CT systems differ in technical specifications and software, significantly impacting radiation dose with shielding. The specific AEC of the manufacturer has a diverse radiation dose increase with patient shields in the overranging zone [12].

#### CT of vulnerable patient groups

Although major societies (e.g., NCRP [24], ACR [34], AAPM [16]) advise against fetal shielding in CT, evidence-based studies are limited (see Table 4). The SSK recommends considering shielding in vulnerable groups. A risk-benefit assessment, as in Table 4, can guide decisions if risks of obscuring or increased dose are addressed and all criteria are met; otherwise, shielding should be avoided [50, 53].

Radiation protection should focus on strict medical indication, precise examination area definition, optimized protocols, correct patient positioning, and minimizing scan lengths. As described by Ryckx et al [65], for instance, a reduction in scan length of up to 3 cm enables a reduction in uterine dose of up to 24% for thoracic CT examinations and up to 47% for the upper abdomen [47, 65].

These strategies are more effective in reducing radiation than focusing solely on patient shielding.

## Discussion

The use of patient shielding in clinical practice remains uncertain due to contradictory and ambiguous recommendations from associations. Some data indicate that patient shielding can increase radiation exposure, highlighting the need for reassessment. While benefits are studied, the actual clinical risks are often underrepresented.

A clear international guideline on patient shielding is needed to eliminate ambiguities and must rely on evidence-based studies to provide thorough recommendations. While such guidelines are desirable, local regulations must be established first. Current research lacks evidence-based data on benefits versus risks, particularly in CT. This requires clearly listing benefits and risks, as in Tables 1–4, and addressing gaps through internal analyses, as done in some studies [28, 29, 39, 66].

In projection radiography and interventional radiology, patient shielding offers marginal benefit and may increase radiation via AEC or repeated exams. Thus, it is not recommended, especially for pediatric patients (Table 1) and pregnant patients (Table 2). In CT, assessing benefits versus risks is more complex due to gaps in research, particularly regarding risk assessment (Tables 3 and 4). Recommendations often rely on phantom measurements that may not account for differences among CT manufacturers. Shields must be used without compromising AEC or image quality, requiring patient and manufacturer-specific consideration. Clinical practice studies are essential to develop evidence-based guidelines regarding the actual risks of artifacts, repeated exams, and shielding-related increases in radiation dose across different CT systems. Evaluating and quantifying increased radiation dose from shielding in patient cohorts is essential.

The SSK allows patient shielding at the patient’s request if it can be implemented without disadvantages [47]. However, medical examinations should always be conducted according to current state-of-the-art standards, rather than patient requests. Instead, the focus should be on patient explanations.

The ALARA principle (“as low as reasonably achievable“) is not only a fundamental principle of radiation protection, but also a central element of patient care. According to article 5 of the Council Directive 2013/59/EURATOM [67] the application of this principle must be based on state-of-the-art science and technology. Decisions should be guided by current scientific and technical progress to ensure radiation use is justified, minimized, and in the patient’s best interest. The interdisciplinary collaboration between physicians, radiographers, and qualified medical physicists is crucial in evaluating shielding’s risk-benefit ratio and ensuring decisions are based on the latest science and technology.

Professional societies must provide clear, evidence-based positions. Targeted campaigns should educate staff and patients, ensuring adequate information. This statement clarifies examination practices, especially for vulnerable groups, and supports long-term implementation. We hope it encourages clinical studies to assess the benefits and risks of patient shielding.

## Conclusion

The current scientific and controversial debate on the use of patient contact shielding in medical imaging shows that traditional approaches or generalized recommendations fail to account for patient- and system-specific variation in technology and patient needs. Evidence-based clinical studies are urgently needed to examine benefits and, in particular, risks in real practice for each specific examination region. These studies have not been conducted yet, which is illustrated in this special report. Filling these gaps is required for the development of precise and patient-specific guidelines, while the current state-of-the-art can be derived from past studies summarized in the following key point statement:

Projection radiography (pediatric radiology):

- For pediatric patients, positioning shields is complex and may lead to repeated imaging due to movement or incorrect placement, especially during pelvic exams.
- Patient contact shielding should not be used in neonatal imaging.

Interventional radiology:

- Shielding is impractical for most angiographic interventions.
- For CT-guided interventions, shielding may benefit patients when safely positioned outside the scan area.

CT:

- For safe use in CT, shields must be placed outside the scan area and be securely fixed to prevent displacement. If these conditions cannot be guaranteed, shielding should not be used.
- A new topogram without shielding is recommended if patient shields are outside the scan area but inside the overranging area to avoid dose increases from the overranging effect.
- If there is doubt about shield placement in the primary beam or overranging area, it is better not to use shielding.
- The priority in radiation protection should focus on strict medical indication, precise examination area definition, optimized protocols, correct patient positioning, and minimizing scan lengths.

## Abbreviations

a.p.: anterior-posterior
AAPM: American Association of Physicists in Medicine
AEC: Automatic exposure control
BIR: British Institute of Radiology
DRL: Diagnostic reference level
ICRP: International Commission on Radiological Protection
lat.: lateral
p.a.: posterior-anterior
SSK: Commission on Radiological Protection (German: Strahlenschutzkommission)
SSRMP: Swiss Society of Radiobiology and Medical Physics
